# Laparoscopic Surgery for Gastric Cancer: The European Point of View

**DOI:** 10.1155/2019/8738502

**Published:** 2019-05-12

**Authors:** Mickael Chevallay, Minoa Jung, Felix Berlth, Chon Seung-Hun, Philippe Morel, Stefan Mönig

**Affiliations:** ^1^Department of Visceral Surgery, Geneva University Hospital, Geneva, Switzerland; ^2^Department of Surgery, University Hospital Köln, Köln, Germany

## Abstract

**Objective:**

Multiple Asian studies have proved the feasibility of laparoscopic approach for surgical treatment of gastric cancer. The difference between Asian and European patients could limit their application in Europe. We reviewed the literature for European studies comparing open gastrectomy with laparoscopic approach in the treatment of gastric cancer.

**Method:**

We searched the keywords gastric cancer and laparoscopy in MEDLINE and EMBASE. We included all studies published between 1990 and 2016 and conducted in Europe.

**Result:**

We found 1 randomized and 13 cohort studies which compared laparoscopic with open gastrectomy. We found no mean difference in the number of lymph nodes harvested between laparoscopic and open group (mean difference: -0.49; 95% CI: -2.42; 1.44, p=0.62) and no difference of short-term or long-term mortality (short-term odds ratio: 0.74, p=0.47; long-term odds ratio: 0.65, p=0.11). We found a longer operative time in the laparoscopic group (mean difference: 35.75 minutes, p<0.01) but lesser reoperation rate than the open group (odds ratio: 1.55 p=0.01).

**Conclusion:**

European based population studies found results comparable with their Asian counterpart. In the current state of evidence, minimally invasive surgery for gastric cancer is safe and can achieve the same oncological results.

## 1. Introduction

Gastric cancer is the 4th cause of cancer-related death in Europe [1]. Major progress has been made in its management as, for example, the introduction of perioperative chemotherapy. Surgery remains the only curative therapy. Improvement has also been made in this particular area. Extent of lymphadenectomy has undergone progressive changes to find the one associated with the best staging and survival. D2 lymphadenectomy is actually recommended as a standard for all TNM stages N+ and above T2 gastric cancer in Japanese guidelines [2]. The next step in surgical development is minimally invasive surgery. The oncological principle with adequate lymphadenectomy and negative resection margin needs to be equivalent as open procedure. Inability to offer the same quality to their patient discourages surgeons to use new techniques without strong evidence. Laparoscopy has already proven its superiority over open surgery for colorectal cancer with shorter hospital stay [3] and similar long-term oncological outcomes [4]. Kitano et al. [5] performed the first laparoscopic distal gastrectomy for gastric adenocarcinoma in 1994 with a modified D1 lymph node dissection. After this preliminary result, the technique has spread in Asia with several cases series confirming its safety and feasibility. The first European experience is published in 1999. Azagra et al. [6] reported a series of 13 gastric cancer cases operated with a laparoscopic approach in Belgium. Majority of the evidence for laparoscopic surgery for gastric cancer comes from Asian studies. Conclusions of this evidence should be extrapolated to the European population with caution. Several differences exist between the Asian and European population: first, European patients have higher BMI. Visceral fat can make the laparoscopic approach more difficult. Second, tumors are more advanced in Europe. Nationwide screening programs in Japan [7] and Korea [8] diagnose early cancer suitable for surgery. Finally, the surgical caseload is higher in Asia with high volume hospital. Surgeons reach the peak of their learning curve faster than a surgeon in low incidence area. In order to synthesize the European evidence, we conduct a systematic review of the European studies comparing laparoscopic and open gastrectomy for gastric cancer.

## 2. Method

### 2.1. Search Strategy, Selection Criteria, and Data Extraction

We searched the keywords “laparoscopic” and “open” and “gastric cancer” and “gastrectomy” in Pubmed, EMBASE, Cochrane Library, and ClinicalTrials.gov databases. Inclusion criteria were European trials that compare open and laparoscopic gastrectomy for gastric adenocarcinoma. We excluded case report, case series, trials nonconducted in Europe, non-English articles, histology other than adenocarcinoma, and robotic approach. One investigator read the complete article when the inclusion criteria were met and extracted the following endpoints for each study: age, gender, tumor localization, number of lymph node retrieved, R0 resection rate, type of gastrectomy, type of lymphadenectomy, type of anastomosis technique, operative time, blood loss, conversion rate, postoperative complication, length of stay, short-term mortality (defined as death occurring less than 30 days after the surgery), recurrence rate, and long-term mortality. This systematic review was conducted according to the Preferred Reporting Items for Systematic Reviews and Meta-Analyses (PRISMA) guidelines [9].

### 2.2. Statistical Analysis

Statistical analysis was performed using the RevMan 5 software (Review Manager (RevMan) [Computer program]. Version 5.3. Copenhagen: The Nordic Cochrane Centre, The Cochrane Collaboration, 2014). We used difference in means for continuous variable and odds ratio for dichotomous variable with 95 % confidence interval (CI). The statistical significance was set for p-value < 0.05. We estimated the heterogeneity for each outcome by calculating the I^2^ value. If the I^2^ value was superior to 25%, we assumed that the studies heterogeneity was high. We used in these cases a random effect meta-analysis model. When the I^2^ value was inferior to 25%, we used a fixed effect meta-analysis model. For each outcome, we assessed publication bias with funnel plots and used Egger's test to detect asymmetry [24]. In cases where values were reported as median, we used the Hozo formula [25] to estimate the corresponding mean and standard deviation.

## 3. Results

### 3.1. Studies Characteristics

**Figure 1:**
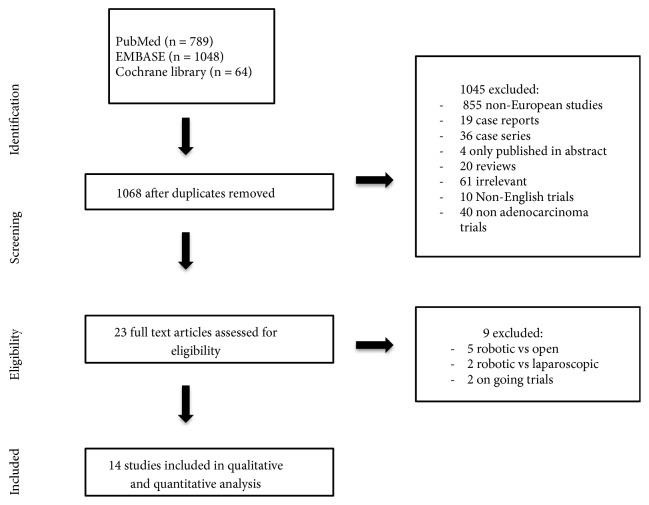
Study selection process.

We found 1068 articles in total corresponding to the search criteria. We excluded 855 non-European studies, 19 case reports, 36 case series, 4 only published in abstract form, 20 reviews, 61 irrelevant articles, 10 non-English trials, and 40 trials which included nonadenocarcinoma tumor. Twenty-three articles met the initial criteria and have been completely read to assess their eligibility. After this process, we excluded 9 articles (5 articles that compared robotic and open approach, 2 that compared laparoscopic and robotic approach, and 2 ongoing clinical studies). We included in the final analysis 14 studies [10–23].  Figure 1 resumes the study selection process. Studies are summarized in Tables 1, 2, and 3. All studies were conducted between 1992 and 2016. One was a randomized clinical trial [11] and the others were retrospective or prospective nonrandomized trials. The number of patients in the studies ranged from 29 [14] to 10713 [22]. 7 were conducted in Italy [11, 12, 16, 18–21], 3 in United Kingdom [14, 17, 22], 2 in France [10, 15], 1 in Netherland [23], and 1 in Belgium [13].

### 3.2. Population and Tumor Characteristics

Eleven studies [11, 13–17, 19–21, 23] reported mean age of patients. There was no mean difference of age between laparoscopic and open group in the different studies (mean difference: -0.17; 95% confidence interval: -1.12 to 0.77, p=0.72) with low heterogeneity (I^2^: 0%). Nine studies [10, 11, 13, 16, 18, 19, 21–23] reported patients gender. We found that more male patients were included in the open approach groups with an odds ratio OR=1.27 (95% CI:1.06 to 1.52, p=0.01) with low heterogeneity (I^2^=17%).

Only two studies reported the number of patients which received neoadjuvant treatment: chemotherapy [14] or chemoradiotherapy [23].

Tumor localization was reported in 6 studies [13, 18–21, 23]. The odds ratio for distal tumor comparing open and laparoscopic approach was nonsignificant (OR= 0.81; CI 0.64 to 1.01, p=0.07) with low heterogeneity (I2: 0%).

### 3.3. Histological Results

One study [17] included only early gastric cancer defined as T1-T2 cancer. The others studies treated all stage of gastric cancer.

Number of lymph nodes harvested was reported in 12 studies [10–12, 14–21, 23].

**Figure 2:**
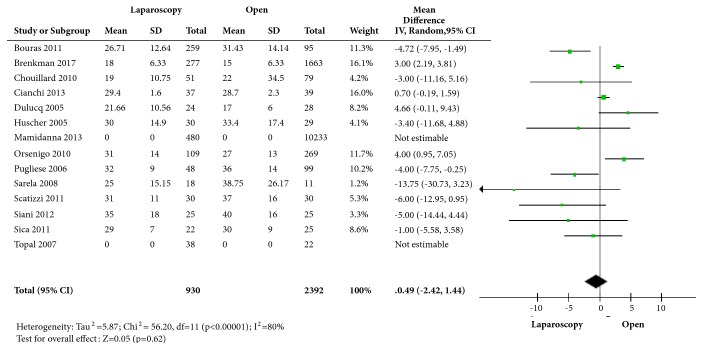
Comparison of the mean difference for lymph node resected in each study.

We found no significant mean difference in the number of lymph nodes harvested between laparoscopic and open group (mean difference: -0.49. 95% CI: -2.42; 1.44, p=0.62) with a high heterogeneity (I^2^=80%) (Figure 2).

We analysed the subgroup of studies that specified to perform a D2 lymphadenectomy [12–16, 18, 20, 23]. Mean difference was also nonsignificant (mean difference: -1.24. 95% CI: -4.78 to 2.30. p=0.49) with high heterogeneity (I^2^=78%).

The adequacy of resection margin was described in 7 studies [10, 12–16, 18, 21, 23]. We compared the rate of negative resection margin between the open and laparoscopic approach. We found an odds ratio favoring the open approach (OR= 1.58, 95% CI 1.10; 2.28, p=0.01) with a low heterogeneity (I^2^=0%).

### 3.4. Operative Results

Partial and total gastrectomy was performed in 8 studies [10, 12, 14–16, 19, 21, 23].

The proportion of subtotal gastrectomy was 61% (n=1371) in the open group and 39% (n=369) in the laparoscopic group. Two studies [11, 17] included only partial gastrectomy and two studies only total gastrectomy [13, 20]. Four studies [11, 17, 19, 21] performed either D1 or D2 lymphadenectomy according to the tumor stage. In 8 studies, D2 lymphadenectomy was the only lymph node dissection performed [12–16, 18, 20, 23]. Two studies did not report their type of lymphadenectomy [10, 22].

Twelve studies reported their anastomosis techniques after gastrectomy: seven studies performed a Roux-en-Y reconstruction [12–18], three studies [10, 11, 19] performed either Roux-en-y or Billroth II, and one study performed either Roux-en-Y or Billroth I [17].

**Figure 3:**
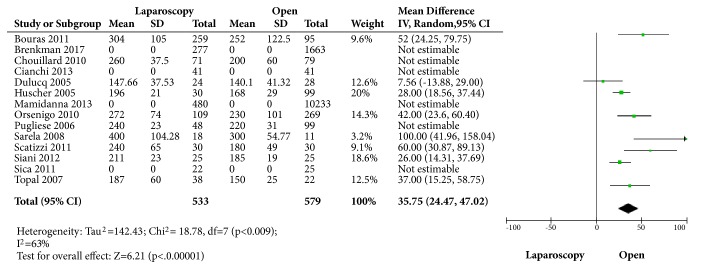
Comparison of the mean difference of operative time in each study.

Ten studies [10–18, 20] described the mean operative time and it was longer in the laparoscopic group with a mean difference of 35.75 minutes (95% CI: 24.47 to 47.02, p<0.001) with high heterogeneity (I^2^=63%). (Figure 3)

Eight studies [11–13, 15–17, 20, 21] reported the mean volume of blood loss. Mean difference was in favor of the laparoscopic approach with -182.96 millilitres (95% CI: -232.02 to -133.91) p<0.001 with high heterogeneity (I^2^= 88%). Conversion rate was described in 9 studies [11, 12, 14–16, 18, 20, 21, 23]. 51 conversions were needed in total of these studies, which included 629 laparoscopic gastrectomies with a rate of 8% conversion rate.

Concerning the learning curve, one study [17] found no difference for lymph node retrieved before the 60th and after the 90th cases (respectively, 29.38 versus 26.19) and another one [13] showed less operative time in the last 19 cases of their series (180 versus 210 minutes, p=0.005). Brenkman et al. [23] showed that, after 10 procedures, the conversion rate decreased from 13 to 2% (p=0.001) and the lymph node retrieval increased from 18 to 21 nodes (p=0.045).

### 3.5. Postoperative Results

Length of stay was reported in 10 studies [10–12, 14–16, 18, 20–22]. Mean difference between open and laparoscopic gastrectomy was 4.10 days (95%CI: -4.87 to -3.33, p<0.001) with a high heterogeneity (I^2^=87%).

Number of duodenal leakage was described in 6 studies [10–12, 14, 16, 21]. Odds ratio comparing laparoscopy with open surgery for the rate of duodenal leakage favors the minimally invasive approach (OR: 3.10; 95% CI 1.70; 5.66, p=0.0002) with a low heterogeneity (I^2^=0%). When we excluded the study from Orsenigo et al. with the higher rate of duodenal leakage, the odds ratio became nonsignificant (OR: 1.79; 95% CI: 0.61; 5.26, p=0.29) with low heterogeneity (I^2^=0%).

**Figure 4:**
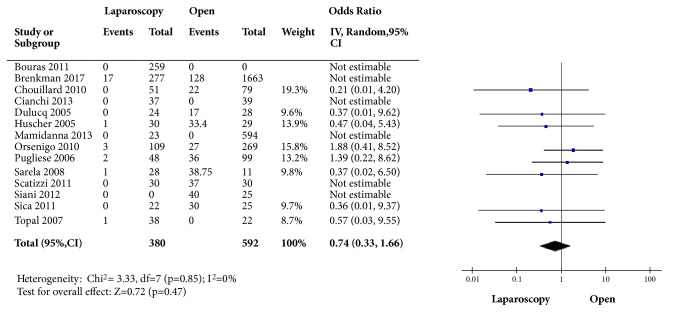
Odds ratio for in-hospital mortality comparing open and laparoscopic approach.

Anastomotic leakage was reported in 8 studies [11–13, 17–21]. We found an odds ratio without statistically significance (OR: 1.25; 95% CI: 0.50; 3.10, p=0.64) with low heterogeneity (I^2^=0%) when comparing laparoscopic to open approach. Odds ratio for reoperation was calculated using the data from 8 studies [10, 12–14, 16, 18, 21, 22] and was found to favor the laparoscopic approach (OR: 1.55; 95% CI: 1.09;2.20, p=0.01) with mild heterogeneity (I^2^=35%). In-hospital mortality was reported in 13 studies. The odds ratio comparing laparoscopic and open procedure for short-term mortality was nonsignificant (OR: 0.74; 95% CI: 0.33 to 1.66; p=0.47) with low heterogeneity (I^2^=0%) (Figure 4).

### 3.6. Overall and Oncological Survival

We found an expression of mean follow-up period in 5 studies [10, 11, 15, 17, 18]. The shorter follow-up was 18 months in both groups [18] and the longer was 49 and 52 months in the open and laparoscopic group, respectively [11].

When we compared the mean follow-up in the laparoscopic and open procedure, we found no difference in mean (mean difference: -2.22; 95% CI: -5.65 to 1.22, p=0.21 ) with I^2^=36%. Number of oncological recurrences (local or metastatic) was described in 4 studies [10, 11, 17, 19]. The odds ratio was nonsignificant between the two groups (odds ratio=0.48, 95% CI: 0.19 to 1.25, p=0.13) with I^2^=35%.

Long-term mortality, defined as mortality occurring later than 30 postoperative days, was reported in 8 studies [10, 11, 15, 17–20, 23]. We found no odds ratio significant between the two groups (OR: 0.65, 95%CI: 0.39 to 1.09, p=0.11) with I^2^=0%.

## 4. Discussion

Minimally invasive surgery is the next step in gastric cancer surgical management. More than half of the worldwide cases of gastric cancer are diagnosed in East Asia (China, Japan, and Korea) each year [26]. Despite the disadvantage of lower incidence, European countries manage to acquire experience. The studies included in this review totalize 1490 patients treated with laparoscopic approach and this serves as a basis for European evidence. The European studies confirm the superiority of laparoscopic procedures with less blood loss and a shorter hospitalization. Li et al. [27] showed in their meta-analysis, which included randomized controlled trials mostly Asian (13 for only 1 European), a mean difference of 100.20 mL (95%CI, - 131.68 to - 68.72, I^2^=90%) in blood loss and -0.84 in hospital stay (95% CI, -1.35 to -0.32; I^2^=76%). Xiong et al. [28] in another meta-analysis with 15 nonrandomized clinical trials showed less blood loss -201.19 mL (95%CI: -296.50 to -105.87; I^2^ = 98%) and a significantly shorter postoperative hospital stay -3.55 (95%CI: -5.13--1.96, P < 0.0001) in the laparoscopic group. Advantages of laparoscopic procedure are confirmed in the European population and are consistent with the Asian findings. Operative time follows the same pattern. In our review, we found a mean difference of 30 minutes between laparoscopic and open procedure. In the two meta-analyses, Li et al. [27] and Xiong et al. [28] showed also a higher mean operative time in laparoscopic group, respectively, mean difference of 48.25 and 68.96 minutes. Minimally invasive surgery has a longer operative time due to technical difficulties. Surprisingly, the mean difference of operative time is lower in European studies. This can be explained by the expansion of laparoscopic indication in upper gastro-intestinal pathologies. Even in low case volume of gastric cancer, laparoscopic experience can be obtained through other indications. One example is the use of minimally invasive surgery in the management of infracarinal oesophageal carcinomas. Historically, oesophagectomy was performed with Ivor-Lewis procedure with open approach: laparotomy and right-thoracotomy. An emerging approach is hybrid minimally invasive oesophagectomy in which the abdominal step is conducted by laparoscopy [29]. The laparoscopic approach can be applied to certain stage of the intervention while maintaining the adequacy of oncological resection at the thoracic stage.

Gastrectomy for gastric cancer should respect oncological principles such as a minimal number of lymph node retrieval and negative resection margin. The review of the different studies conducted in Europe comparing laparoscopic and open procedure showed no difference in lymph node retrieval especially when D2 lymphadenectomy was performed. The work from Li et al. [27] and Xiong et al. [28] showed also no mean difference between number of lymph node during open and laparoscopic approach (respectively, mean difference: -1.27 (95% CI: -3.03 to 0.49) and -2.49 (95%CI: -5.18 to 0.21)).

Since 1997, the American Joint Committee for Cancer (AJCC) [29] proposed a minimal number of 15 dissected nodes for the accurate prognosis of N status. The exact number of lymph nodes has been since questioned. The authors of an international retrospective study [30] have concluded that improvement in survival was obtained when an optimum of 29 lymph nodes retrieval was achieved. Regardless of the surgical approach, the lymph node goal should be this number.

In our experience, minimally invasive approach was associated with higher risk of positive margin than open procedure. Asian meta-analysis did not report the rate of positive resection margin. This result should be moderate with the fact that only half of the studies reported their resection margin rate. A difference exists between West and East: Western guidelines [31] propose a resection margin of 4-5 cm whereas the Japanese Gastric Cancer Association [32] ranges the adequate margins from 2 to 5 cm according to the T status. One explanation is that the plateau of gastrectomy learning curve in Europe was not reached during these studies. Resection margin is an area of improvement for European minimal invasive surgery.

The rate of duodenal leakage was higher in the open group in European studies. However, one study [16] had a high rate of leakage. The author hypothesis for this high rate of duodenal leakage was that they did not use a running suture for the duodenal stump. When this study was excluded from the analysis, the open and laparoscopic approach did not differ in terms of duodenal and anastomosis leakage.

A cutoff for the learning curve was not set by European studies. In an observational study, Kim et al. [32] showed that improvement on surgical parameters and postoperative course was seen after a cutoff value of 46 cases. Improvement of operative time was also seen after 50 cases in another observational study [33].

They are limitations to our review. The studies were different in their designs: only one was a randomized clinical trial and the others were retrospectives. Methods of the studies were not standardized with multiples types of reconstruction and lymphadenectomy. Neoadjuvant chemotherapy was reported in only two studies and this could have been another bias. Finally, the male gender was overrepresented in the open approach group and this could have led to introduce a selection bias.

For future direction, two European trials are being conducted. The first, LOGICA-trial [34], from Netherlands, is a multicenter, randomized clinical trial comparing laparoscopic and open gastrectomy for gastric cancer (ClinicalTrials.gov identifier: NCT02248519). The second, STOMACH-trial [35], is also a multicenter, randomized clinical trial comparing laparoscopic and open gastrectomy but after neoadjuvant chemotherapy and is international (Germany, Netherland, Spain, United Kingdom) (ClinicalTrials.gov Identifier: NCT02130726).

## 5. Conclusion

Laparoscopic procedure for gastric cancer in Europe causes less blood loss, and shorter hospital stays with similar number of lymph nodes harvested. These European results are similar to their Asian counterparts. Asian evidence remains stronger with more randomized clinical trials. In order to fill this gap, two Europeans trials are ongoing to determine the best surgical approach in the era of neoadjuvant treatment.

## Figures and Tables

**Table 1:** General summary of the studies: year of publication, country, type of design, and number of patients (LC: laparoscopic group, SD: standard deviation, and N/E: nonexistent).

Study	Publication year	Inclusion years	Country	Type of study	Number of patients: open	Number of patients: laparoscopy (LC)	Gender female, N (%)	Age, mean (SD)	Gastrectomy total or partial
Dulucq et al. [10]	2005	1995-2004	France	Prospective non randomized trial	28	24	Open: 16 (57%) LC: 14 (58%)	Open: 68.82 (14.61) LC: 72.33 (10.8)	Total: Open 11, LC 8 Partial: Open 17, LC 16

Huscher et al. [11]	2005	1992-1996	Italy	Randomized trial	29	30	Open: 8 (27%) LC: 12 (40%)	Open: 63.6 (13.2) LC: 63.2 (12.5)	Partial

Pugliese et al. [12]	2006	2000-2005	Italy	Retrospective	99	48	N/E	N/E	Total: Open 35, LC 5 Partial: Open 64, LC 43

Topal et al. [13]	2007	2003-2006	Belgium	Retrospective	22	38	Open: 5 (23%) LC: 15 (39%)	Open: 69 (12) LC: 68 (12)	Total

Sarela et al. [14]	2008	2005-2007	United Kingdom	Retrospective	11	28	N/E	N/E	Total: Open 7 LC 6 Partial: Open 4, LC 12

Chouillard et al. [15]	2010	2001-2007	France	Retrospective	79	51	N/E	Open: 66.1 (13.2) LC: 61.6 (14.2)	Total: Open 30, LC 44 Partial: Open 49, LC 37

Orsenigo et al. [16]	2010	2002-2008	Italy	Retrospective	269	109	Open: 100 (37%) LC: 53 (49%)	Open: 66.73 (12) LC: 66.57 (11)	Total: Open 171, LC 17 Partial: Open 98, LC 92

Bouras et al. [17]	2011	2000-2009	United Kingdom	Retrospective	95	259	N/E	Open: 64.9 (12) LC: 65.1 (13.5)	Partial

Scatizzi et al. [18]	2011	2006-2009	Italy	Retrospective matched cohort	30	30	Open: 16 (53%) LC: 14 (47%)	Open: 69 (10.75) LC: 70 (12)	Partial

Sica et al. [19]	2011	2000-2004	Italy	Prospective non randomized	25	22	Open: 12 (48%) LC: 9 (41%)	Open: 68 (5.25) LC: 67 (9.25)	Total: Open 7, LC 5 Partial: Open 18, LC 17

Siani et al. [20]	2012	2003-2009	Italy	Prospective non randomized	25	25	N/E	Open: 66 (7.8) LC: 65 (8.5)	Total:

Cianchi et al. [21]	2013	2008-2012	Italy	Retrospective matched cohort	41	41	Open: 16 (39%) LC: 17 (41%)	Open: 74 (13.75) LC: 73 (10)	Total: Open 29, LC 29 Partial: Open 12, LC 12

Mamidanna et al. [22]	2013	2000-2010	United Kingdom	Retrospective	10233	480	Open: 3502 (34%) LC: 207 (43%)	N/E	N/E

Brenkman et al. [23]	2017	2010-2014	Netherlands	Retrospective	1663	277	Open: 628 (38%) LC: 104 (37%)	Open: 68.4 (11.9) LC: 68.5 (11.5)	Total: Open 554, LC 137 Partial: Open 1109, LC 140

**Table 2:** Summary of the studies: operative results (LC: laparoscopic group, SD: standard deviation, and N/E: nonexistent).

Study	Type of anastomosis	Type of Lymphadenectomy, number (%)	Mean number of lymph node, mean (SD)	R0 rate, number (%)	Operative time, mean (SD)	Blood loss, mean (SD)	Conversion rate, number (%)	Duodenal leakage, number (%)	Anastomosis leakage
Dulucq et al. [10]	Roux-en-Y or Billroth II	N/E	Open: 17 (6) LC: 21.66 (10.56)	Open: 27 (96%) LC: 24 (100%)	Open: 140.10 (41.32) LC:147.66 (37.53)	N/E	N/E	Open: 0 LC:1 (4%)	N/E

Huscher et al. [11]	Roux-en-Y or Billroth II	D1: Open 9 (31%), LC 9 (30%) D2: Open 20 (69%), LC 21 (70%)	Open: 33.4 (17.4) LC: 30 (14.9)	N/E	Open: 168 (29) LC: 196 (21)	Open: 391 (136) LC: 229 (144)	0 (0%)	Open: 1 (3.4%) LC: 0	Open: 0 LC: 0

Pugliese et al. [12]	Roux-en-Y	D2	Open: 36 (14) LC: 32 (9)	Open: 99 (100%) LC: 48 (100%)	Open: 220 (31) LC: 240 (23)	Open: 394 (125) LC: 150 (85)	1 (2%)	Open: 1 (1%) LC: 2 (4%)	Open: 2 (2%) LC: 0 (0%)

Topal et al. [13]	Roux-en-Y	D2	N/E	Open: 21 (95%) LC: 37 (97%)	Open: 150 (25) LC: 187 (60)	Open: 175 (337.5) LC: 20 (98.75)	N/E	N/E	Open: 0 LC: 2 (5%)

Sarela et al. [14]	Roux-en-Y	D2	Open: 38.75 (26.17) LC: 25 (15.15)	Open: 7 (63%) LC: 13 (72%)	Open: 300 (54.7) LC: 400 (104.2)	N/E	5 (28%)	Open: 1 (9%) LC: 3 (17%)	N/E

Chouillard et al. [15]	Roux-en-Y	D2	Open: 22 (34.5) LC: 19 (10.75)	Open: 73 (92.4%) LC: 49 (96.1%)	Open: 200 (60) LC: 260 (37.5)	Open: 372 (208.75) LC: 150 (205)	2 (3.9%)	N/E	N/E

Orsenigo et al. [16]	Roux-en-Y	D2	Open: 27 (13) LC: 31 (14)	Open: 239 (89%) LC: 103 (94%)	Open:230 (101) LC: 272 (74)	Open: 372 (401) LC:170 (199)	17 (15.6%)	Open: 14 (5.2%) LC: 20 (18.3%)	N/E

Bouras et al. [17]	Roux-en-Y or Billroth I	D1: Open 51 (54%) LC 233 (90%) D2: Open 44 (46%) LC 26 (10%)	Open: 31.43 (14.14) LC: 26.71 (12.64)	N/E	Open: 252 (122.5) LC: 304 (105)	Open: 310.2 (555) LC: 84.9 (198.75)	N/E	N/E	Open: 3 (3%) LC: 7 (3%)

Scatizzi et al. [18]	Roux-en-Y	D2	Open: 37 (16) LC: 31 (11)	Open: 30 (100%) LC: 30 (100%)	Open: 180 (49) LC: 240 (65)	N/E	2 (6.6%)	N/E	Open: 0 LC:2 (7%)

Sica et al. [19]	Roux-en-Y or Billroth II	D1: Open 2 (8%), LC 2(9%) D2: Open 23 (92%), LC 20 (91%)	Open: 30 (9) LC: 29 (7)	N/E	N/E	N/E	N/E	N/E	Open: 0 LC:0

Siani et al. [20]	Roux-en-Y	D2	Open: 40 (16) LC: 35 (18)	N/E	Open: 185 (19) LC: 211 (23)	Open: 495 (190) LC: 250 (150)	0 (0%)	N/E	Open: 0 LC:1 (4%)

Cianchi et al. [21]	Roux-en-Y and billroth II	D1: Open 2 (5%), LC 4 (10%) D2: Open 39 (95%), LC 37 (10%)	Open: 28.7 (2.3) LC: 29.4 (1.6)	Open: 40 (97.6%) LC: 41 (100%)	N/E	Open: 312.4 (42.9) LC: 118.7 (10.7)	0 (0%)	Open: 2 (4.9%) LC: 2 (4.9%)	Open: 2 (4.9%) LC: 0

Mamidanna et al. [22]	N/E	N/E	N/E	N/E	N/E	N/E	N/E	N/E	N/E

Brenkman et al. [23]	N/E	D2	Open: 15 (6.5) LC: 18 (6.33)	Open: 1447 (87%) LC: 249 (90%)	N/E	N/E	24 (9%)	N/E	N/E

**Table 3:** Postoperative results of each study (LC: laparoscopic group, N/E: nonexistent).

Study	Length of stay in days, mean (SD)	Post-operative mortality, number (%)	Follow-up, months, mean	Recurrence, number (%)	Long term mortality, number (%)
Dulucq et al. [10]	Open: 24.6 (9.61) LC: 16.3 (4.73)	Open: 1 (3.6%) LC: 0	Open: 43 (25) LC: 29.4 (20.2)	Open: 8 (28.5%) LC: 1 (4.1%)	1 year Open: 8 (28.5%) LC: 1 (4.1%)

Huscher et al. [11]	Open: 14.5 (4.6) LC: 10.3 (3.6)	Open: 2 (6.9%) LC: 1 (3.3%)	Open: 49.7 (5.2) LC: 52.2 (26.5)	Open: 10 (34.5%) LC: 11 (36.6%)	5 years Open: 21 (72.4%) LC: 21 (70%)

Pugliese et al. [12]	Open: 18 (5) LC: 10 (3)	Open: 3 (3%) LC: 2 (4.2%)	N/E	N/E	N/E

Topal et al. [13]	N/E	Open: 1 (4.5%) LC: 1 (2.6%)	N/E	N/E	N/E

Sarela et al. [14]	Open: 31.75 (22.95) LC: 22.5 (15.98)	Open: 1 (9%) LC: 1 (5.5%)	N/E	N/E	N/E

Chouillard et al. [15]	Open: 11.5 (6.5) LC: 8 (4.5)	Open: 3 (3.8%) LC: 0	Open: 33 (10.8) LC: 29 (10.3)	N/E	3 years Open: 45 (57%) LC: 31 (60.7%)

Orsenigo et al. [16]	Open: 15 (12) LC: 13 (9)	Open: 4 (1.4%) LC: 3 (2%)	33 both groups	N/E	N/E

Bouras et al. [17]	N/E	Open: 0 LC: 1 (0.4%)	Open: 44.1 (27.9) LC: 43.3 (30.2)	Open: 2 (0.8%) LC: 1 (1.1%)	3 years Open: 1 (1.1%) LC: 2 (0.8%)

Scatizzi et al. [18]	Open: 9 (4.25) LC: 7 (11)	Open: 0 LC: 0	Open: 18 (8.7) LC: 18 (8.7)	N/E	1 year Open: 13 (43.3%) LC: 9 (30%)

Sica et al. [19]	N/E	Open: 1 (4%) LC: 0	N/E	Open: 13 (52%) LC: 11 (50%)	3 years Open: 14 (56%) LC: 12 (54.5%)

Siani et al. [20]	Open: 14.5 (3.1) LC:10.5 (1.5)	N/E	Open: 32.6 LC: 31.9	N/E	Open: 12 (48%) LC: 11 (44%)

Cianchi et al. [21]	Open: 11.5 (0.8) LC: 8.1 (0.5)	Open: 2 (4.9%) LC: 1 (2.4%)	N/E	N/E	N/E

Mamidanna et al. [22]	Open: 14 (1.33) LC: 11 (1.5)	Open: 594 (5.5%) LC: 23 (4.8%)	N/E	N/E	N/E

Brenkman et al. [23]	N/E	Open: 128 (8%) LC: 17 (6%)	N/E	N/E	1 year Open: 365 (22%) LC: 61 (22%)
